# Acoustic Measures of Brazilian Transgender Women's Voices: A Case–Control Study

**DOI:** 10.3389/fpsyg.2021.622526

**Published:** 2021-05-31

**Authors:** Anna Paula Villas-Bôas, Karine Schwarz, Anna Martha Vaitses Fontanari, Angelo Brandelli Costa, Dhiordan Cardoso da Silva, Maiko Abel Schneider, Carla Aparecida Cielo, Poli Mara Spritzer, Maria Inês Rodrigues Lobato

**Affiliations:** ^1^Gender Identity Program, Universidade Federal do Rio Grande do Sul, Programa de Pós Graduação em Psiquiatria e Ciências do Comportamento, Porto Alegre, Brazil; ^2^Identity Program, Universidade Federal do Rio Grande do Sul, Programa de Pós Graduação em Ciências Médicas: Endocrinologia, Porto Alegre, Brazil; ^3^Pontifícia Universidade Católica do Rio Grande do Sul, Programa de Pós-Graduação em Psicologia e do Programa de Pós-Graduação em Ciências Sociais, Porto Alegre, Brazil; ^4^Psychiatry & Behavioural Neurosciences, Faculty of Health Sciences, McMaster University, Hamilton, ON, Canada; ^5^Department of Psychiatry and Behavioural Neurosciences, McMaster University, Hamilton, ON, Canada; ^6^Speech Therapy Department, Universidade Federal de Santa Maria, Santa Maria, Brazil

**Keywords:** voice, acoustic analysis, gender dysphoria, speech therapy, transgender (LGBT)

## Abstract

**Objective:** This study aims to compare the acoustic vocal analysis results of a group of transgender women relative to those of cisgender women.

**Methods:** Thirty transgender women between the ages of 19 and 52 years old participated in the study. The control group was composed of 31 cisgender women between the ages of 20 and 48 years old. A standardized questionnaire was administered to collect general patient data to better characterize the participants. The vowel /a/ sounds of all participants were collected and analyzed by the Multi-Dimensional Voice Program advanced system.

**Results:** Statistically significant differences between cisgender and transgender women were found on 14 measures: fundamental frequency, maximum fundamental frequency, minimum fundamental frequency, standard deviation of fundamental frequency, absolute jitter, percentage or relative jitter, fundamental frequency relative average perturbation, fundamental frequency perturbation quotient, smoothed fundamental frequency perturbation quotient, fundamental frequency variation, absolute shimmer, relative shimmer, voice turbulence index (lower values in the cases), and soft phonation index (higher values in the cases). The mean fundamental frequency value was 159.046 Hz for the cases and 192.435 Hz for the controls.

**Conclusion:** Through glottal adaptations, the group of transgender women managed to feminize their voices, presenting voices that were less aperiodic and softer than those of cisgender women.

## Introduction

According to the Diagnostic and Statistical Manual of Mental Disorders-5 (American Psychiatric Association., 2013), gender dysphoria is characterized by a marked incongruence between the experienced/expressed gender and the primary and/or secondary sexual characteristics, usually accompanied by the desire to make the body as congruent as possible with the preferred sex through surgery and hormonal treatment (WHO, 2004).

“Transgender” is a generic term that encompasses a wide spectrum of people who do not identify with the sex to which they were assigned at birth (Ansara and Hegarty, 2012). For these individuals, the inconsistency between their gender identity and the sex attributed to them at birth creates discomfort (Schneider et al., 2017).

Psychological processes can mediate the relationship between minority stress and mental health, although data relating these factors to trans women are scarce, the degree of satisfaction of this population with their own body is directly related to mental health outcomes (Kanamor and Xu, 2020). In a study that used a minority stress model to explore the indirect effects on the association between attacks based on transphobia and anxiety and depression through the degree of body satisfaction of a person, it was seen that body satisfaction mediated the relationship between violence based on transphobia and mental health. Clinical intervention that promotes body satisfaction, including access to gender-confirming therapies, voice therapy, and especially hormonal therapy, can prevent negative mental health outcomes among trans women (Klemmer et al., 2021).

Acoustic voice analysis has become a primary tool to assess and quantify the vocal quality of an individual. It is frequently used to compare pre- and posttreatment vocal quality. The Multi-dimensional Voice Program advanced system (MDVPA) is a software used to perform acoustic voice analysis that quantifies the glottal source signal (Amir et al., 2009). The parameters commonly analyzed are measures of fundamental frequency, frequency disturbance, amplitude disturbance, and noise measurements (Sørensen et al., 2016). The reliability and validity of these analyses depend on several factors, such as the microphone type, data acquisition system, ambient noise levels, sampling rate, software used, and quality of the collected vocal sample (Lovato et al., 2016).

The frequency disturbance measurements provided by the MDVPA are Jita, Jitt, RAP, PPQ, sPPQ, and vf0. The measures of amplitude perturbation are ShdB, Shim, APQ, sAPQ, and vAm. The noise measurements are NHR, VTI, and SPI. The MDVPA voice wrap measures are identified through DVB and NVB. Measures of unvoiced segments are obtained by NUV and DUV, and measures of subharmonic components are obtained by NSH and DSH (Finger et al., 2009; Susana Finger et al., 2009).

In their efforts to feminize their voice, some transgender women engage in vocal compensation, such as performing laryngeal elevation during speech (Misołek et al., 2016), making instinctive adaptations of the vocal tract (Schneider et al., 2017), or seeking specialized procedures potentially involving surgery and/or speech therapy. Although an increase in fundamental frequency (f0) leads to a more feminine voice, a higher f0 is often not enough for the voice to be perceived consistently as a female voice (Hoffmann et al., 2017; Schwarz et al., 2017). Studies have noted that the voice to be recognized as female needs to have a f0 between 155 and 160 Hz (Spencer, 1988; Dacakis, 2002); another study showed that transgender women had their voices recognized as feminine when their f0 was between 164 and 199 Hz (Gelfer and Schofield, 2000). However, both verbal and nonverbal factors arising from communication are extremely relevant to the new gender identity (Gray and Courey, 2019).

McNeill et al. (2008) concluded that, in transgender women, satisfaction with their own voice was not directly related to f0 but rather to the self-perception and perception of listeners. In a study conducted with 20 transgender women, it was found that the speakers who considered their voices to be more feminine were judged as more feminine by listeners, which shows the importance of exploring patients' perceptions of their own voice (Hancock et al., 2010). In this sense, knowing the acoustic vocal characteristics of trans women will provide support to the professional speech therapist to develop and provide strategies for vocal feminization, in addition to modifying the f0, which help trans women to perceive themselves as female, reinforcing the desired gender role.

A previous study described aspects of perceptual–auditory vocal analysis and the f0 of 32 transgender women and compared these qualities to those of 28 cisgender women without vocal complaints. The study concluded that transgender women exhibited a f0 between 80 and 150 Hz more frequently than cisgender women did (*p* = 0.003). Furthermore, transgender women had hypernasal resonant focus (*p* < 0.001) and roughness (*p* = 0.031) more frequently than cisgender women did (Schwarz et al., 2017).

Another study used inverse filtering of the airflow signal to indirectly assess vocal fold function in 13 transgender women. The participants were asked to sustain the vowel /a/ first in her biologically male voice and then again in her female voice. The perceptual ratings of a feminine voice were associated with a f0 of 180 Hz or greater, although f0 did not differ significantly between male and female voice production (Gorham-Rowan and Morris, 2006).

Being recognized by weavers according to their type of experience is essential for this population. The trans population wants changes in all characteristics that are not congruent with their gender identity and expression. Voice can be considered a characteristic of gender expression, as the general population tends to attribute gender according to what is seen and heard, that is, when a trans woman is able to feminize her voice, she feels more comfortable with her gender expression, which can affect factors such as self-esteem and satisfaction with herself, thus being able to ease the effects of minority stress experienced by that population. Studies on the vocal production of trans people are important because the voice is one of the fundamental elements of social recognition and gender expression. The current study aims to compare the results of acoustic vocal analysis of the glottal source between a group of transsexual women and a group of cisgender women to investigate the differences between the vocal parameters of the two groups.

## Materials and Methods

### Design

This is a prospective case–control study. Because the present study involved a database search, records with incomplete data were excluded.

### Setting and Participants

The institution's ethics committee approved this study (number 14075). All participants were informed regarding the procedure and signed the informed consent prior to participating in the research, according to Resolution 466/12 from the National Commission of Ethics in Research. Data were analyzed from subjects in the database whose information was collected from January 2015 to July 2016.

Protig is the name by which the Gender Identity Program of the Hospital de Clínicas de Porto Alegre became known. A pioneer in Brazil and created in 1998, it was one of the first spaces created for transgender people to be entitled to the treatment of gender claims and surgeries free of charge. The program plays an important role in assisting transgender people with gender dysphoria in Brazil. Through a multidisciplinary team, it provides care to individuals who seek clinical service in order to minimize suffering between the incongruity of their physical characteristics with their gender identity.

**Figure 1:**
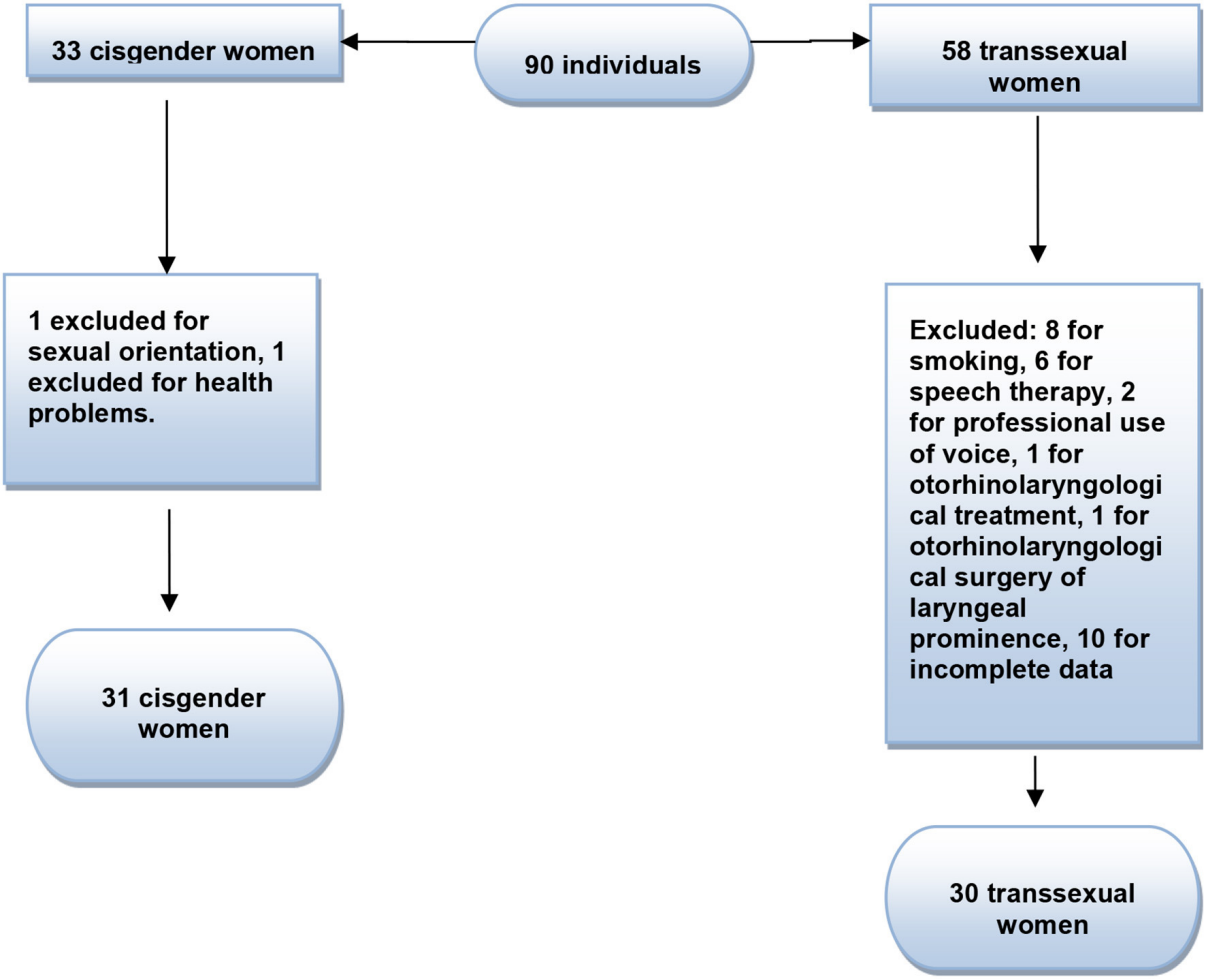
Sample flow chart.

The initial sample of the studies was composed of 58 trans women who consulted at PROTIG/Hospital de Clínicas de Porto Alegre (HCPA), who presented with the diagnosis of gender dysphoria already established, and who accepted the invitation to participate in the study. The participants were located at the PROTIG outpatient clinic and were invited to the research. The difference between the assistance provided by the service and the participation in the research was duly explained, which would not affect its treatment and would be voluntary. After agreeing with and signing of the free and informed consent form, the participants were directed to the Clinical Research Center. Sociodemographic data were collected from the participants after applying the exclusion criteria (Figure 1). The sample of trans women participating in the study was 30, all of whom had their voices analyzed. The inclusion criteria were as follows: transgender women between the ages of 18 and 55 and who had not undergone surgery or speech therapy for vocal feminization. The exclusion criteria were as follows: smoking, report of current use of illicit substances and alcohol in excess, hearing loss (assessed by auditory screening), report of illness that could interfere in the efficiency of vocal production, professional use of voice, voice feminization or reduction of laryngeal prominence, and psychiatric or neurologic conditions that could interfere with the participants' understanding of the study tasks. The control group was composed of 31 cisgender women volunteers without pre-established vocal pathologies (Figure 1). The inclusion criteria were as follows: women aged 18–55 years and women who self-identified as cisgender. The exclusion criteria were as follows: smoking, current use of illicit substances and/or self-report of alcohol abuse, hearing loss (assessed by auditory screening), disease related to or alterations in speech-articulatory structures that could interfere with the efficiency of vocal production (such as respiratory or digestive problems), professional use of voice, speech or otorhinolaryngological treatment, and psychiatric or neurologic conditions that could interfere with the participants' understanding of the study tasks.

### Clinical Voice Assessment

All volunteers completed a standardized questionnaire that included identification data, use of medications, vocal complaints, presence of disease that could affect the efficiency of vocal production, professional use of voice, and previous phonotherapeutic or otorhinolaryngological treatment. In the same questionnaire, there were two questions about vocal perception: “What do you think about your own voice?” and “What do others think of your voice?” An evaluation of the phonoarticulatory structures was carried out to assess chewing and swallowing difficulties.

All transgender women had been using gender-affirming hormonal treatment for at least 9 months to up to 20 years. Two individuals reported that they self-medicated before being followed up at the endocrinology team of PROTIG at the HCPA.

Additionally, an auditory screening was performed with an audiometer inside an audiometric booth using meatoscopy to discard possible earwax plugs and/or other visually perceptible audiological changes, with frequencies ranging from 500 to 4,000 Hz in 25-dB steps (Interacoustics, Ad229). Individuals who presented with acoustic alterations were referred for complimentary otorhinolaryngological evaluation. A screening of phonoarticulatory structures was also performed to rule out possible alterations that could compromise vocal assessment, such as poorly adapted dental prostheses and substantial respiratory changes.

### Acoustic Analysis

The vocal recordings took place inside audiometric cabins with ambient noise lower than 50 dB NPS, which are located at the Speech and Hearing Unit of HCPA. A professional microphone (Behringer, ECM 8000) and a digital recorder (Zoom, H4n) were used to record voice samples. The participants were instructed to produce a sustained vowel /a/ emission three times, with a distance of 4 cm and a 90° angle between the mouth and the microphone. Five seconds of the vowel /a/ emission was used for the acoustic analysis. The first 2 s of the beginning of the emission was excluded, while the analyzed recording was still ensured to be at least 5-s long, even after the emission. The beginning of the emission was excluded for two reasons. First, vocal attack interference was avoided in the data analysis (Hoffmann et al., 2017). Second, voice instabilities are typically perceived in the first 2 s of voice emission, which may also interfere in the data analysis.

Acoustic vocal glottic source analysis was performed using MDVPA (Kay PENTAX®). This software allows for the extraction of measures of acoustic voice characteristics that enable the analysis of signal and vowel frequency: f0, f0 maximum (fhi), f0 minimum (flo), standard deviation of f0 (STD), absolute jitter (Jitta), percentage jitter (Jitt), relative mean of the disturbance (RAP), pitch disturbance quotient (PPQ), smoothed pitch disturbance quotient (sPPQ), variation of f0 (vf0), shimmer in dB (ShdB), percentage shimmer (Shim), amplitude perturbation quotient (APQ), smoothed amplitude perturbation quotient (sAPQ), amplitude variation (vAm), noise-harmonic ratio (NHR), turbulence index of the voice (VTI), soft phonation index (SPI), degree of vocal breaks (DVB), degree of subharmonic segments (DSH), degree of non-voiced segments (DUV), number of vocal breaks (NVB), number of subharmonic segments (NSH), and number of segments voiced segments (NUV). All of these variables were analyzed in subsets according to the parameter selected, based on the fact that there is still no exact match between a given acoustic measure and a specific characteristic of the phonological physiology (Gorham-Rowan and Morris, 2006; Finger et al., 2009; Janaína da Silva Berto et al., 2009; Beber and Cielo, 2010; Roman-Niehues and Cielo, 2010; Schwarz et al., 2017).

For this study, f0 values of 150 to 250 Hz were typically considered female, while f0 values between 80 and 150 Hz were typically considered male (Guimarães, 2003). For the other measures, the range of normal values proposed by the MDVPA was used as a standard of comparison for the subjects of both groups.

### Statistical Analysis

Considering the non-normal distribution of the extracted variables pertaining to cisgender and transgender women's voices, a non-parametric Mann–Whitney test and a Wilcoxon test were used to compare the mean values between groups. The significance threshold was *p* < 0.05. Analyses were performed by comparing the group of transsexual women with the group of cisgender women.

## Results

The ages of the control group ranged from 19 to 48 years, with a mean age of 32.2 years (*p* = 0.0401). The ages of the transgender group ranged from 19 to 52 years, with a mean age of 34.69 years and an average hormonal treatment of 54 months. No patient had received sexual reassignment surgery.

Statistically significant differences between cisgender and transsexual women were found for f0, Fhi, Flo, STD, Jita, Jitt, RAP, PPQ, sPPQ, vf0, ShdB, Shim, VTI (lower values in the cases), and SPI (higher values in the cases) (Table 1).

**Table 1:** Comparisons of numerical variables between cisgender and trans women.

**Variable**	**Cisgender women**		**Transsexual women**		*p*-value^*^	**Threshold**
	**Average**	**SD**	**Average**	**SD**		
Age	32.20	8.27	34.69	9.59	0.401	
Fhi (Hz)^*^	212.335	268.41	169.933	188.50	0.001	
Flo (Hz)^*^	172.431	245.31	148.959	253.80	0.001	
STD (Hz)^*^	4.682	5.435	3.476	7.301	0.001	2.115
Jita (us)^*^	70.137	42.670	55.987	48.246	0.034	83.200
Jitt (%)^*^	1.354	0.857	0.864	0.687	0.002	1.040
RAP (%)^*^	0.818	0.510	0.518	0.415	0.002	0.680
PPQ (%)^*^	0.792	0.527	0.504	0.425	0.002	0.840
sPPQ (%)^*^	1.029	0.727	0.870	0.828	0.048	1.020
vf0 (%)^*^	2.390	2.568	2.268	4.942	0.002	1.100
ShdB (dB)^*^	0.410	0.140	0.353	0.190	0.022	0.350
Shim (%)^*^	4.571	1.411	4.014	2.119	0.023	3.810
APQ (%)	3.164	9.316	2.933	14.640	0.085	3.070
sAPQ (%)	5.933	2.928	5.425	2.295	0.318	4.230
vAm (%)	21.740	9.7274	17.660	73.226	0.112	8.200
NHR	0.142	0.27	0.140	0.23	0.728	0.190
VTI^*^	0.203	0.854	0.044	0.15	0.041	0.061
SPI^*^	7.364	3.992	10.567	4.528	0.003	14.120
DVB (%)	0.089	3.395	0	0	0.147	1.000
DSH (%)	0.213	3.469	0.777	2.309	0.014	1.000
DUV (%)	0.430	2.149	0.330	1.182	0.696	1.000
NVB	0.16	0.64	0	0	0.168	0.900
NSH	0.345	0.564	0.128	0.380	0.014	0.900
NUV	0.65	3.41	0.55	1.97	0.600	0.900
f0 (Hz)	192.435	184.36	159.04	184.36	0.001	

* *p < 0.05*.

*SD, Standard deviation; fhi, f0 maximum; flo, f0 minimum; STD, standard deviation of f0; Jitta, absolute jitter; Jitt, percentage jitter; RAP, relative mean of the disturbance; PPQ, pitch disturbance quotient; sPPQ, smoothed pitch disturbance quotient; vf0, variation of f0; ShdB, shimmer in dB; Shim, shimmer percentual; APQ, amplitude perturbation quotient; sAPQ, smoothed amplitude perturbation quotient; vAm, amplitude variation; NHR, noise-harmonic ratio; VTI, turbulence index of the voice; SPI, soft phonation index; DVB, degree of vocal breaks; DSH, degree of subharmonic segments; DUV, degree of non-voiced segments; NVB, number of vocal breaks; NSH, number of subharmonic segments; NUV, number of non-voiced segments; f0, fundamental frequency, Hz, Heartz*.

The questionnaire about vocal perception showed that, in relation to the first question, 33.33% of transgender women answered that they like their own voice, 13.33% answered that they find their voices neutral, and 53.33% answered that they do not like their own voices. In relation to the second question, 60% answered that listeners think that their voices sound feminine, 13.33% answered that their voices are evaluated as neutral, and 26.66% answered that their voices are recognized as masculine by listeners (Villas-Boas et al., 2019).

## Discussion

In this study, transgender women performed vocal adaptations that interfere with their vocal production and their vocal acoustic measures. The most commonly used acoustic measures in studies are f0, frequency and amplitude disturbance, noise, vocal tremor, voice breaks, and subharmonic components (Araújo et al., 2002). In a study with English speakers, Oates and Dacakis (Oates and Dacakis, 1983) report an average male f0 of 128 Hz, with a minimum f0 of 60 Hz and a maximum of 260 Hz. In female voices, the average f0 is 227 Hz, with a minimum f0 of 128 Hz, a maximum f0 of 520 Hz, and an ambiguous gender range from 128 to 260 Hz. A study carried out in a Portuguese-speaking Brazilian population concluded that f0 values from 150 to 250 Hz were considered to be typically female, while f0 values between 80 and 150 Hz were considered to be typically male (Guimarães, 2003). A study carried out in China with Cantonese speakers who had their gender analyzed by means of vocal samples showed that there was 75% correct gender identification at 162.01 and 204.97 Hz for male and female stimuli, respectively (Poon and Ng, 2014).

The mean f0 found in the case group was 159 Hz, which was lower than that in the control group but within the f0 range considered to constitute female vocal standards. It should be taken into account that the case group in the present study did not receive speech therapy or surgical interventions involving the larynx, so this elevation of f0 reaching the female range was obtained by means of instinctive adaptations or favorable anatomy (Ansara and Hegarty, 2012; Oates and Dacakis, 2015; Misołek et al., 2016; Schneider et al., 2017) (Table 1). This factor may also be related to body size (Leung et al., 2018) and more feminine facial characteristics (Misołek et al., 2016). Self-performed adaptations by transgender women should be stimulated more systematically and technically correctly by voice professionals since they are not muscular adaptations that cause overload to the speech apparatus, as not all adaptations are healthy from a physiological point of view (Dacakis, 2002).

Noise measures report altered voices. The main measures are NHR, SPI, VTI, SPI, and HNR (Beber and Cielo, 2010). In the present study, the SPI was found to be within the normal range for both groups (Table 1). However, the SPI value of the cases was greater than that of the controls. The SPI measure indicates noise at high frequencies, possibly related to breathiness (González et al., 2002), which may be a factor present in the voices of cases (but not controls) caused by attempts at feminization involving the use of breath to soften the male emission.

A previous study was carried out to evaluate the contribution of VTI and SPI acoustic parameters to vocal evaluation through the GRBASI scale in a group of 94 cisgender women and men with and without vocal complaints and who were between 19 and 81 years old. It was observed that the higher the VTI value was, the greater the general degree of vocal deviation, roughness, and tension. In relation to SPI, the higher the breathiness was, the lower the tension (Galdino de et al., 2013). The SPI measure indicates noise at high frequencies, possibly related to breathiness (González et al., 2002; Karlsen et al., 2020), which may be a factor present in the voices of cases (but not controls) caused by attempts at feminization involving the use of breath to soften the male emission. On the other hand, the SPI result confirms data from the literature suggesting that the measure is sensitive to soft closure of the vocal folds (Galdino de et al., 2013). Considering that, in the present study, VTI was higher in the control group and SPI was higher in the case group, we can hypothesize that the voices of cisgender women present greater noise due to turbulence (VTI) than the voices of transsexual women do. The difference is most likely justified by transgender women's increased breathiness/softness (SPI) compared to that of cisgender women's voices (Table 1).

In a study aiming to investigate the acoustic differences between the voices of a control group and a group of patients presenting different types of pathologies of the larynx, it was found that the mean NHR of the control group was 0.14%, while that of patients with laryngeal pathologies varied from 0.18 to 0.22%, depending on the pathology in question (González et al., 2002). In a study conducted with groups of choral singers and non-singers, higher NHR values were found in the group of non-singers (0.07%) than in the group of singers (0.04%) (Ravi et al., 2019). Given that the high end of the normal range for NHR in the MDVPA is 0.190%, the NHR values obtained in the present study (0.140% for cases and 0.142% for controls), which showed no significant difference between groups, are within the normal range. Only one previous study highlighted the NHR as the least reliable variable of the program in question (Leong et al., 2013).

One study suggested the following normal standard values for voices of male subjects without vocal pathologies: Jitt, 0.66%; RAP, 0.37%; Shim, 3.23%; APQ, 2.59%; VTI, 0.06%; and SPI, 8.88% (Sørensen et al., 2016). In the present study, the group of transgender women, whose vocal anatomy is male, showed average values closer to male than to female standard values (Table 1). Furthermore, the mean values for the acoustic measures of the case group were higher than those of the normal MDVPA range for males for the variables STD, vf0, shdB, shim, sAPQ, vAm, DSH, DUV, and NSH and higher than the reported female range values in the previously cited study.

While the cycle-to-cycle frequency disturbance is called jitter, the disturbance of the cycle-to-cycle amplitude of a vocal sample is called shimmer (Beber and Cielo, 2010). The present study showed higher Jitt, RAP, PPQ, and sPPQ in the controls, with jitter being higher in the control group than in the transgender women group. The ShdB, Shim, APQ, sAPQ, and vAm measurements were related to shimmer and were also higher in the control and case groups, but shimmer was higher in the control group than in the case group. Considering that the shimmer demonstrates phonatory stability and that its high values reflect greater noise in the emission, that is, breathy voices (Carvalho-Teles and Rosinha, 2008), it can be concluded that both groups present breathiness in the voice. However, it was observed that in both groups the ShdB, Shim, sAPQ, and vAm measurements were above the normal MDVPA range and that in the controls the APQ measurement also exceeded this limit.

In other words, all jitter and shimmer values in the control group were higher than those in the transgender women, and all shimmer values were above the normal range of MDVPA for both groups (Table 1). These results indicate the alteration of the glottic signal for both cases and controls, suggesting aperiodicity in both groups and a higher breathiness index (shimmer measurements) in the controls than in the cases. However, the cases also presented with breathiness, a fact reinforced by the SPI. In contrast to previous reports (Finger et al., 2009), the voices of cisgender women had greater aperiodicity (jitter and shimmer) than the transgender women's voices did.

In a study carried out on 35 adult euphonic individuals, including 24 women and 11 men, with the objective of measuring shimmer parameters, it was shown that the women had the following values: Shim = 2.22%; APQ = 1.75%; sAPQ = 3.30%; vAm = 7.04%. The observed values for male voices were as follows: Shim = 2,892%; APQ = 2.61; sAPQ% = 3.43; vAm = 6.38% (Oates and Dacakis, 2015). In the present study, for the same variables mentioned above, the controls and cases presented the following values: Shim = 4.57%; APQ = 3.16%; sAPQ = 5.93%; vAm = 21.745% and Shim = 4.14%; APQ = 2.93%; sAPQ = 5,425%; vAm = 17.667%, respectively. Taking into account that euphonic individuals are those who present a voice without alterations (Nicastri et al., 2004), we can affirm that, on average, the voices of both the cases and the controls in the present study are altered compared to those in the cited study. It should also be considered that the APQ of the group of cases was closer to the APQ value of the male group, evidencing similarity in this measure of shimmer between the transgender women's voices and the cisgender men's voices.

Part of the case group of the present study participated in a study in 2017 in which they answered the transsexual voice questionnaire for male-to-female transsexual. The questionnaire included aspects of vocal satisfaction and how the voice interfered with personal and professional relationships, such as discrimination. Most participants had low scores. However, many experienced stress because they were perceived as the opposite gender or demonstrated dissatisfaction with their voice (Schwarz et al., 2016).

Internalized transphobia is an individual form of minority stress that occurs when trans people develop negative perceptions of themselves in response to repeated exposure to social stigma against trans people and is directly linked to the poor mental health of that population (Testa et al., 2015; Staples et al., 2018). A study conducted with a population of Korean trans men and women showed that Korean transgender adults face similar public health concerns such as high prevalence of depressive symptoms, suicidal ideation, and suicide attempts, which were directly correlated with internalized transphobia experienced by this population (Lee et al., 2020). So, a voice consistent with the gender experienced can reduce the stigma imposed by society on a trans woman, which can be beneficial for the mental health of this population.

## Conclusion

Based on the results obtained in the present study, it was concluded that transgender women perform vocal adaptations that interfere with their vocal production. This finding suggests that transgender women have lowest f0 and less aperiodic and softer voices than cisgender women have.

This suggests that, throughout their lives, they make muscular adaptations and speech projections that result in voices that sound feminine without necessarily undergoing surgical interventions and speech therapy.

The objective vocal characteristics found are also relevant to understand and help trans women to achieve vocal passability not based only on f0. For many trans women, vocal feminization with change of f0 is very difficult or is not possible for anatomo-physiological reasons. Thus, through adaptations and vocal malleability and without surgical interventions, the studied group obtained vocal identification congruent with the experienced gender expression, even with a lower f0 than the controls, which increases their social passability and self-esteem.

### Study Limitations

Because the study was conducted only with Brazilian Portuguese-speaking individuals, there are cultural differences in vocal ranges that are learned and imprinted early in life, and this type of analysis is difficult to translate across different languages and cultures. Even though they are not voice professionals, a large part of the sample in the control group was composed of people who use their voice a lot throughout the day, which may have interfered with the parameters found in this group. Studies on the vocal production of trans people are important because the voice provides objective measures in relation to the characteristics that differ between the genders, constituting itself as one of the fundamental elements of social recognition.

## Data Availability Statement

The original contributions generated for the study are included in the article/supplementary material, further inquiries can be directed to the corresponding author/s.

## Ethics Statement

The studies involving human participants were reviewed and approved by Comitê de Ética em Pesquisa do Hospital de Clínicas de Porto Alegre. The patients/participants provided their written informed consent to participate in this study.

## Author Contributions

All authors listed have made a substantial, direct and intellectual contribution to the work, and approved it for publication.

## Conflict of Interest

The authors declare that the research was conducted in the absence of any commercial or financial relationships that could be construed as a potential conflict of interest.
